# Rational, emotional, or both? Subcomponents of psychopathy predict opposing moral decisions

**DOI:** 10.1002/bsl.2547

**Published:** 2021-10-20

**Authors:** Nicole Claire Hauser, Craig S. Neumann, Julia Marshall, Andreas Mokros

**Affiliations:** ^1^ Department of Forensic Psychiatry University Hospital of Psychiatry Zurich Zurich Switzerland; ^2^ Department of Psychology University of North Texas Denton Texas USA; ^3^ Department of Psychology Boston College Chestnut Hill Massachusetts USA; ^4^ Department of Psychology Personality and Forensic Psychology and Diagnostics Division University of Hagen Hagen Germany

**Keywords:** cognition‐emotion, deliberate, empathy, PCL‐R facets, psychopathy, sacrificial moral dilemmas

## Abstract

Recent research has documented a small but significant correlation between psychopathic capacities and utilitarian moral judgment, although the findings are generally inconsistent and unclear. We propose that one way to make sense of mixed findings is to consider variation in perspective‐taking capacities of psychopathic individuals. With this in mind, we had criminal offenders (*n* = 60), who varied in their psychopathy levels according to the Psychopathy Checklist‐Revised (PCL‐R), respond to common sacrificial moral dilemmas (e.g., trolley dilemmas) under different conditions. In a baseline condition, participants simply responded to the sacrificial moral dilemmas as is typically done in previous research. In an “emotion‐salient” condition, participants had to reason about the emotions of another person after solving moral dilemmas (deliberative processing). In the “emotion‐ambiguous” condition, participants saw images of people in distress, after solving moral dilemmas, but did not have to explicitly reason about such emotions (spontaneous processing). The four PCL‐R facets predicted distinct interference effects depending on spontaneous versus deliberative processing of hypothetical victim's emotions. The findings suggest that the use of a multi‐faceted approach to account for cognitive and moral correlates of psychopathy may help address previously mixed results. Implications and future directions for theory and research are discussed.

## 1 INTRODUCTION

Psychopathic individuals are characterized by a lack of empathy and remorse, deceitful and manipulative traits, and a profound disregard for social rules that often lead to antisocial, law‐breaking behavior (Hare, 2003; Hare & Neumann, 2008). Their affective callousness in combination with social deviance is often considered the epitome of immoral behavior (Glenn et al., 2010; Leistico et al., 2008). Psychopathic criminal offenders are frequently presented as extreme examples of immoral persons, and a lack of empathy has been argued to be a primary reason for their deficient moral compass (Blair, 2007).

### 1.1 Utilitarian versus deontological decisions

Despite psychopathic individuals' antisocial behavior, a recent meta‐analysis found only a small relationship between non‐standard moral judgment and psychopathic traits, suggesting that perhaps psychopathic individuals may only exhibit subtle differences in moral judgment in comparison to less psychopathic individuals (Marshall et al., 2018). The most common way of measuring moral judgment and decision‐making involves sacrificial moral dilemmas (Greene et al., 2001; Thomson, 1986). In these dilemmas, participants are typically presented with an impending problem, and they have to decide whether to sacrifice some people to save a larger number of people. For example, in the classic trolley dilemma, a trolley is headed to kill five individuals, but the participant can sacrifice one person by administering a switch to stop the trolley to save the five individuals, or avoid sacrificing the one person, which leads to the five individuals on the track dying. In a more personal variation of the trolley scenario, the footbridge dilemma, instead of administering a switch, the participant can push a large man off a footbridge to stop the train from killing five individuals instead of one person (Greene et al., 2004).

The choice to sacrifice one life to save many demarks a *utilitarian* judgment, which stands for a calculative decision solely based on an evaluation of the consequences of an action. In contrast, the choice to avoid sacrificing one person to save many demarks a *deontological* decision, which represents the appreciation of moral duties and obligations that are inherent to certain actions (e.g., one shall not kill; the end does not justify the means). While both scenarios, the trolley and the footbridge dilemma, involve the sacrifice of one life for many, most people experience a moral difference between the two dilemma types (Greene et al., 2001). Thus, the more personally experienced footbridge dilemma has been linked to stronger emotional responses on a neural level, resulting in predominantly deontological decisions in healthy individuals (Greene et al., 2001). In comparison, the impersonal trolley dilemma has been associated with more utilitarian decisions (Cushman et al., 2006).

Many psychologists and philosophers have argued that making deontological decisions—the choice to avoid sacrificing a life for the greater good—involves intuitive and emotional responding. Utilitarian decisions in contrast, are considered more rational and unemotional (Greene et al., 2001, 2008). This has been referred to as a dual‐process model of moral reasoning (Greene et al., 2008; Reynolds & Conway, 2018). In support of the model, research has shown that participants often will make more deontological decisions under time pressure and also individuals who score lower on cognitive reflection tasks often make more deontological decisions (Paxton et al., 2012). What complicates this picture is that psychopathic individuals—who are characterized by immoral actions and antisociality—have been documented, in some studies, to make more utilitarian decisions in comparison to individuals not elevated on psychopathic traits (Glenn et al., 2010; Kahane et al., 2015; Koenigs et al., 2007, 2012). At the same time, various studies reported no such associations between psychopathy and utilitarian decision‐making (Cima et al., 2010; Pletti et al., 2017).

### 1.2 Judging versus acting

One explanation for these heterogeneous relationships is that psychopathic individuals may be able to distinguish between “right” and “wrong,” but are insufficiently motivated to act in accordance with such a moral understanding (Cima et al., 2010; Luke et al., 2021; Marshall et al., 2017), perhaps in part, due to differences in cognitive‐affective processes. In support of this, psychopathy has been linked to utilitarian choices of action but not to corresponding utilitarian moral judgments (Pletti et al., 2017; Tassy et al., 2013). Consistent with the suggestion that psychopathy is associated with lack of motivation to act along moral principles, recent evidence suggested that psychopathic individuals experience moral conflicts as emotionally less unpleasant than individuals low in psychopathy (Blair, 2007; Pletti et al., 2017; Seara‐Cardoso et al., 2012).

In this context, research on the cognitive and affective correlates of psychopathy can potentially shed light on the moral “sensibility” of psychopathic individuals. For example, when attending to emotion information, psychopathic individuals are able to respond to it as indicated by fear‐potentiated startle in the presence of threat‐related stimuli (Baskin‐Sommers et al., 2012; Newman et al., 2010). Psychopathic individuals do not appear, however, to incorporate emotional information into further decision‐making processes. For example, research has shown psychopathic individuals display intact momentary regret but deficient avoidance of regret in counterfactual decisions (Baskin‐Sommers et al., 2016).

### 1.3 Spontaneous versus deliberate information processing

The studies drawing a distinction between moral judgments and choices of actions provide evidence that psychopathic individuals might recognize moral matters but that they do not act in accordance with such intellectual recognition. However, such studies raise questions concerning the deliberation of moral decision‐making and the behavior of psychopathic individuals. On that note, additional research may help further our understanding of the cognitive‐affective processes involved in psychopathy and their link to moral reasoning. In tasks that explicitly demand taking the perspective of others, for instance, individuals with elevated psychopathic traits perform similarly to those without elevated traits, indicating adequate theory of mind capacities (Blair et al., 1996; Jones et al., 2010; Shamay‐Tsoory et al., 2010). In contrast, on tasks that do not explicitly demand taking the perspective of others, psychopathic individuals do considerably worse than individuals without elevated traits (Blair, 1995; Drayton et al., 2018).

One way to make sense of these findings is to make a distinction between spontaneous versus deliberative processing of cognitive and emotional information. Evidence for this processing distinction in psychopathic individuals has been found for several empathy correlates, such as moral evaluations (Yoder et al., 2015), emotional empathy (Meffert et al., 2013), and theory of mind (Drayton et al., 2018). When it comes to deliberative reasoning, psychopathic individuals succeed in empathizing with others as indicated by altered brain activation (Meffert et al., 2013; Yoder et al., 2015). In spontaneous reasoning, however, psychopathic individuals have considerable difficulties. Specifically, while an automatic, unintentional representation of others' thoughts and feelings is natural to most people (Apperly, 2010), psychopathic individuals seem to lack this ability, despite the capability to do so deliberately (Drayton et al., 2018). These findings have been interpreted in terms of selective attention abnormalities in information processing of psychopathic individuals. The general idea is that these individuals process goal‐relevant information with a myopic focus and are insensitive to goal‐irrelevant stimuli (Baskin‐Sommers et al., 2011). With regard to moral decisions, this distinction suggests that psychopathic individuals may not generally avoid emotion‐driven decisions, but that they fail non‐deliberately to integrate emotional aspects of complex moral situations into decision‐making processes.

## 2 CURRENT INVESTIGATION

Although research has identified differences between deliberate and spontaneous moral judgments among psychopathic individuals in a variety of domains, less research has been devoted to how previous findings can be translated to a behavioral level of moral decision‐making, particularly moral choices of action. As such, addressing the emotional and cognitive characteristics of psychopathic persons may help further our understanding of their behavior in moral contexts. Specifically, research that incorporates differences in deliberative versus intuitive (spontaneous) emotional processing may shed light on the discrepant research findings on psychopathy and moral decisions. In the current study, we incorporated the distinction between spontaneous versus deliberate emotion processing of empathy‐related content into the investigation of behavioral moral actions. Based on the research discussed, we hypothesized that elevated psychopathic traits would be associated with more utilitarian behavioral choices in sacrificial moral dilemmas. With the incorporation of spontaneous versus deliberate emotion feedback, however, psychopathic traits were expected to be less likely to be associated with utilitarian choices when deliberately processing a potential victim's emotions, compared to a spontaneous processing condition.

More specifically, based on findings that psychopathy is associated with more endorsement of utilitarian choices rather than utilitarian judgment (Pletti et al., 2017; Tassy et al., 2013), we expected psychopathic traits to predict moral choices of actions but not judgments of appropriateness. Additionally, based on previous evidence suggesting that the emotional responses to moral conflicts are lower in psychopathic individuals (Blair, 2007; Pletti et al., 2017; Seara‐Cardoso et al., 2012), we furthermore explored the relationship between psychopathy and self‐reported affective states (valence and arousal) during moral decisions.

Finally, a path analytic approach was employed, given the multifarious and dimensional nature of psychopathy (Hare & Neumann, 2008), and research showing that the different psychopathy traits domains have differential links with external cognitive‐affective and moral correlates (Carré et al., 2013; Schaich Borg et al., 2013). As such, the empirically supported four‐factor model of psychopathy (Neumann et al., 2015) was used to help uncover how certain features of psychopathy are associated with moral reasoning under distinct spontaneous versus deliberative processing conditions.

## 3 METHOD

### 3.1 Participants

The study sample included 60 Caucasian male offenders (*M*_age_ = 39.67, *SD* = 10.28, range = 22–66) from several German correctional and forensic‐psychiatric institutions. Inclusion criteria were defined as broadly as possible with fluent German language skills and the offender status. We included only male offenders for reasons of generalizability (see Efferson & Glenn, 2018, for a review of gender differences in moral, emotional, and cognitive correlates of psychopathy). Hospitalized patients included in the study met additional diagnostic criteria such as substance use disorders (currently abstinent), paraphilia, or severe personality disorders. Participants were excluded if they had clinical diagnoses of schizophrenia or bipolar disorder.

The majority of participants completed main school (56.7%) with lower secondary education (41.7%) or general secondary education (15%). About 16.7% of the participants had completed an apprenticeship, 11.7% were qualified with a university‐entrance diploma, and 3.3% had a University degree. No education was stated by 11.7% of the sample.

For this study, we drew 17 out of the 60 participants from a former study, with previously collected Psychopathy Checklist‐Revised (PCL‐R) scores (Hollerbach et al., 2020). Newly recruited participants received three appointments per person, and the 17 formerly assessed participants received two appointments. Participants were paid 5€ per appointment. Oral and written informed consent was provided by all participants. The University of Zurich Ethics Commission and the cantonal ethics committee of Zurich, Switzerland, as well as the relevant governmental authorities approved the study plan.

### 3.2 Measures

#### 3.2.1 PCL‐R

The German version of the PCL‐R (Mokros et al., 2017) was used to assess psychopathy. With the aid of a semi‐structured interview and extensive institutional file review, this measure involves ratings of 20 items, each on a 3‐point scale (0 = not present, 1 = present to some degree, and 2 = clearly present). The points are scored depending on the degree to which the item applies to the subject with possible total scores between 0 and 40 points. Four subscales divide the items into Interpersonal, Affective, Lifestyle and Antisocial facets, or first‐order Factors (Hare & Neumann, 2008). The Interpersonal facet (i.e., *superficial charm*, *grandiosity*, *pathological lying*, and *manipulative, deceitful skills*) and the Affective facet (i.e., *lack of remorse and guilt*, *shallow affect*, *lack of empathy*, and *failure to accept responsibility*) load on a superordinate Factor 1 mapping psychopathic core personality traits (Hare & Neumann, 2008; Mokros et al., 2017). The Lifestyle facet (i.e., *proneness to boredom*, *impulsivity*, *irresponsibility*, *parasitic lifestyle*, and *lack of realistic long‐term goals*) and the Antisocial facet (i.e., *poor behavioral control*, *early conduct problems*, *juvenile delinquency*, *revocation of conditional release*, and *criminal versatility*) load on a superordinate Factor 2 that maps social deviance.

The PCL‐R is considered as the gold standard for the assessment of psychopathy in forensic samples for its well‐established reliability and validity (Hare, 2003; Mokros et al., 2017). For the German‐language version, construct validity was established in terms of convergent validity with self‐reported psychopathic traits, whereas discriminant validity was ascertained in comparison with broad personality traits (the Big Five), impulsivity, or alexithymia, for instance (Hollerbach et al., 2020).

In the present study, internal consistency estimates for the PCL‐R total score and its Interpersonal and Affective facets were good and acceptable for the Lifestyle facet and the Antisocial facet (see Table 2). All PCL‐R raters were trained by an accredited PCL‐R instructor and achieved excellent interrater reliabilities of 0.97 for PCL‐R total scores for a subsample of 21 randomly selected subjects (indicated with intraclass correlation coefficients in a one‐way random effects model on single measures; ICC). The ICC values for the facet scores were good for the Affective facet (0.77), the Lifestyle (0.86) and the Antisocial (0.87) facets, and excellent for the Interpersonal (0.92) facet (see Koo & Li, 2016). Participants in this study displayed a PCL‐R mean score of 23.03 (*SD* = 9.30), and a range between 4 and 37.90, covering almost the whole spectrum of PCL‐R scores. Half of the participants in our sample had a PCL‐R score of 25 or more, which indicates a high degree of psychopathic traits according to the German manual of the PCL‐R (Mokros et al., 2017). About 38.3% of the participants showed PCL‐R scores of 20 and less, presenting with low to moderate psychopathic traits.

#### 3.2.2 Moral dilemmas

A total of 22 sacrificial dilemmas were shown to the participants. The dilemmas were drawn from various published and widely used sets of moral scenarios (Greene et al., 2001; Moore et al., 2008; Thomson, 1986; Unger, 1996). With respect to Greene et al. (2001) distinction of sacrificial dilemmas into personal versus impersonal dilemmas, we only used personal dilemmas in this study. In personal or “up‐close” dilemmas, social emotions (e.g., concern for others) have been found to play a crucial role for evoking moral conflict in sacrificial dilemmas (Koenigs et al., 2007).

A further subcategorization of moral dilemmas is the division into low‐conflict versus high‐conflict dilemmas (see, e.g., Koenigs et al., 2007). Participants in our study rated the moral dilemmas along a conflict scale. This was because we used dilemmas from different studies not all of which included a conflict rating in order to make sure that participants in our study experienced a moral conflict in the dilemmas presented. Thus, participants were asked to rate how difficult the decision was from 0 to 7 (with 7 indicating an extremely difficult decision). Moral dilemmas with a median of 1 or less qualified for the low‐conflict group with participants rating 7 out of the 22 scenarios as extremely easy to decide on (i.e., low conflict). The remaining 15 dilemmas were considered high‐conflict scenarios, evoking a moral conflict to at least a minimal amount for the majority of the participants and were used for subsequent analyses.^1^

#### 3.2.3 WMT‐II

We controlled for IQ level in our analyses, given that several studies indicated that utilitarian decision‐making is associated with higher working memory capacities (Bartels, 2008; Moore et al., 2008). Additionally, reduced moral capacities in highly psychopathic individuals have been formerly related to differences in IQ levels entirely (O'Kane et al., 1996). And also more generally, intelligence has been found to be associated with variation in psychopathic individuals' performance on cognitive emotional tasks (Olderbak et al., 2018). For the assessment of fluid intelligence, we used the short language‐free matrices test WMT‐2 (*Wiener‐Matrizen‐Test‐2*; Formann et al., 2011). The study sample exhibited a mean IQ of 85.22 (*SD* = 18.72, range 54–121), which is lower than an average IQ in the general population but representative for a forensic sample considering the lower education levels in prison samples (de Tribolet‐Hardy et al., 2014; Olderbak et al., 2018; Schwartz et al., 2015; Ttofi et al., 2016).

### 3.3 Procedure

**FIGURE 1:**
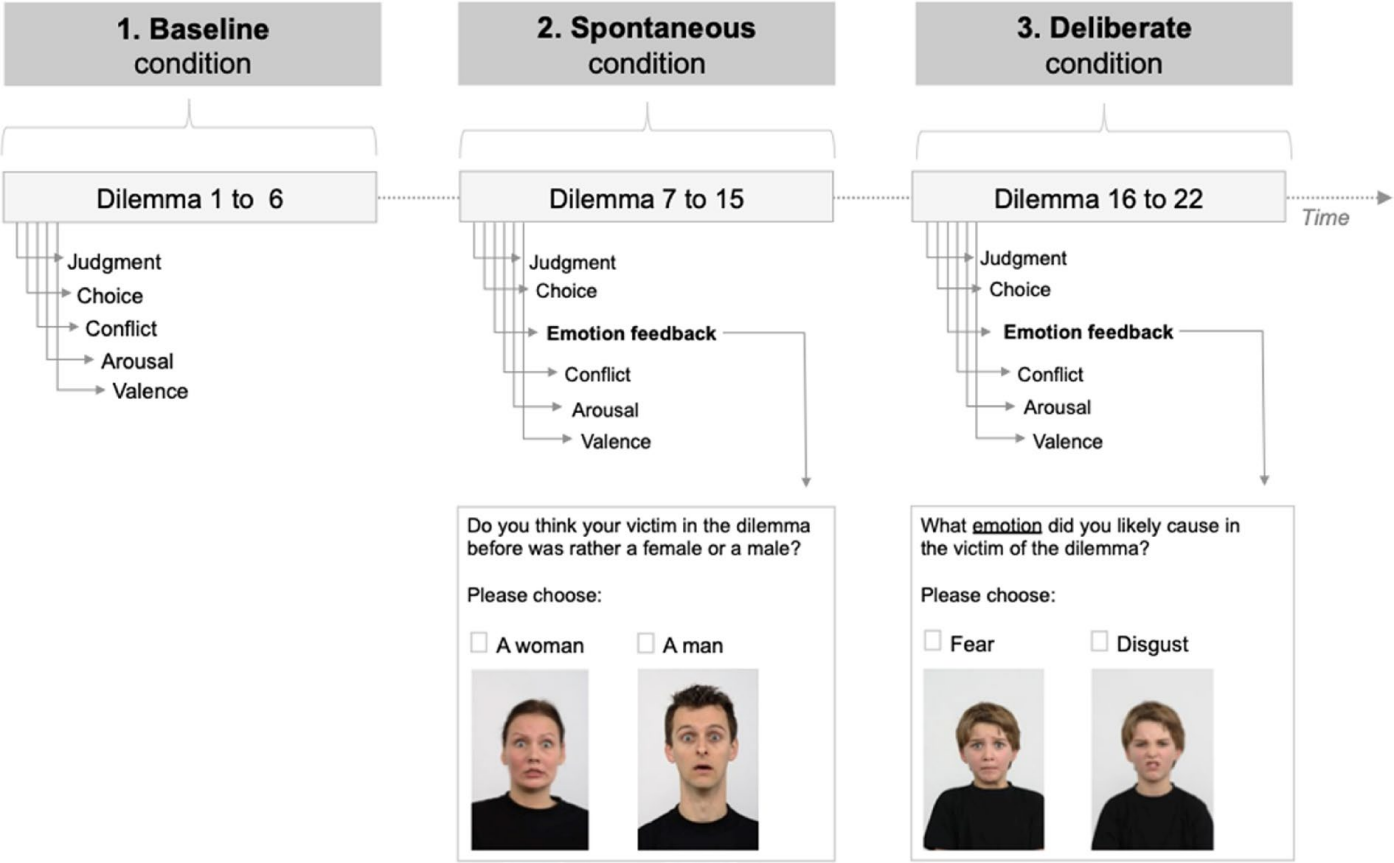
Trial structure with experimental conditions. In the spontaneous and the deliberate condition additional emotion feedback was demanded. While the emotion was peripheral to the participants' focus in the spontaneous condition by asking what gender the victims might have been, the deliberate condition demanded the focus on the emotion of the potential victim

Participants were instructed to read through the dilemmas and then respond to the questions that followed. The dilemmas were presented in text form on a Lenovo Thinkpad T560 (15.60″) with INQUISIT 5 (2016). After a test trial, the moral dilemmas were divided into three task conditions within‐subjects (see Figure 1).

In the first (1), the *baseline* condition, six moral dilemmas were each directly followed by a set of questions. Based on the findings by Pletti et al. (2017), we distinguished between the decision of *moral judgment* and *choice of action* as described above. Participants were asked to first indicate how acceptable they considered the sacrifice of one life for many on a scale from 0 to 7 (moral judgment), and then how likely they would engage in such behavior from 0 to 7 (choice of action). Following the tradition of sacrificial moral dilemma research, utilitarian responses were coded positively (higher values represent higher utilitarian judgments and choices). Participants were then required to rate their arousal from extremely calm (0) to extremely aroused (7) and how pleasant this decision felt (0 indicating extremely unpleasant and 7 indicating extremely pleasant) based on the Arousal and Valence scales of the Self‐Assessment Manikin by Lang et al. (2008). Throughout the questions following the dilemmas, we maintained the same response structure and included the option of zero in order to offer absolute aversion and acceptance levels.

In the second (2) within‐subjects task, the *spontaneous* emotion processing condition, eight new moral dilemmas were presented to the participants that were each again followed by the set of questions described previously. Additionally, after the choice of action, a female and a male face were shown that expressed one of the six basic emotions (i.e., happiness, surprise, sadness, fear, anger, and disgust) along with the question “do you think your victim in the dilemma before was a female or a male?” Pictures with facial expressions were drawn from the Radboud database (Langner et al., 2010). The emotional expression displayed matched the participant's choice of action answer with 0 resulting in a happy face and the replacement of the word *victim* with *person* and 1 to 7 resulting in the display of a negative emotion.

Following the same task structure, in the third task (3), the *deliberate* emotion processing condition, instead of guessing the gender of the victim, participants saw two distinct emotions in a person and were asked to respond to the question “what emotion did you likely cause in the victim of the dilemma?” The emotions again matched the participant's response in the choice of action question and were displayed as text clicks below the pictures. The task structure was tested for feasibility and timing in a pilot study, prior to conducting the study within correctional and forensic‐psychiatric institutions with strict time limits for visitors.

### 3.4 Data analysis

Bivariate correlations were calculated in IBM SPSS statistics version 23 (2015) to assess associations between the key variables. Path models were conducted with Mplus (Muthén & Muthén, 2010), to simultaneously map the four PCL‐R facets onto the decision outcome variables in the three experimental conditions (1—baseline, 2—spontaneous, and 3—deliberate), along with age and IQ as covariates.^2^ Research has shown that social deviance characteristics (Lifestyle, Antisocial PCL‐R scores) and PCL‐R total scores in psychopathic offenders decline at an advanced age, but interpersonal‐affective domains do not show such associations with age (Hare, 2003; Harris et al., 1991; Mokros et al., 2017; Vachon et al., 2013). Thus, age was used along with IQ as a covariate.

Moral decision outcomes in the separate conditions (1), (2), and (3) are represented by moral judgments ratings with higher scores indicating utilitarian judgment (“it is acceptable to sacrifice one for many”) in path model 1, and in model 5 for choice of action ratings (with higher ratings indicating more utilitarian choices; “I'd sacrifice one for many”). For an overview of the models run, see Table 1.

**TABLE 1:** Series of path models run with IVs of PCL‐R facets, covariates, and dependent variables

	Independent variables						Dependent variables		
Model 1	INT	AFF	LIF	ANT	IQ	Age^a^	Judgment (1)	Judgment (2)	Judgment (3)
Model 2	INT	AFF	LIF	ANT	IQ	Age^a^	Judgment (2‐1)	Judgment (3‐1)	‐
Model 3	INT	AFF	LIF	ANT	IQ	Age^a^	Judgment (2‐1)	Judgment (3‐2)	‐
Model 4	INT	AFF	LIF	ANT	IQ	Age^a^	Judgment (3‐2)	Judgment (3‐1)	‐
Model 5	INT	AFF	LIF	ANT	IQ	Age^a^	Choice (1)	Choice (2)	Choice (3)
Model 6	INT	AFF	LIF	ANT	IQ	Age^a^	Choice (2‐1)	Choice (3‐1)	‐
Model 7	INT	AFF	LIF	ANT	IQ	Age^a^	Choice (2‐1)	Choice (3‐2)	‐
Model 8	INT	AFF	LIF	ANT	IQ	Age^a^	Choice (3‐2)	Choice (3‐1)	‐
Model 9	INT	AFF	LIF	ANT	IQ	Age^a^	Total judgments	Total choices	‐
Model 10	INT	AFF	LIF	ANT	IQ	Age^a^	Valence (1)	Valence (2)	Valence (3)
Model 11	INT	AFF	LIF	ANT	IQ	Age^a^	Valence (2‐1)	Valence (3‐1)	‐
Model 12	INT	AFF	LIF	ANT	IQ	Age^a^	Valence (2‐1)	Valence (3‐2)	‐
Model 13	INT	AFF	LIF	ANT	IQ	Age^a^	Valence (3‐2)	Valence (3‐1)	‐
Model 14	INT	AFF	LIF	ANT	IQ	Age^a^	Arousal (1)	Arousal (2)	Arousal (3)
Model 15	INT	AFF	LIF	ANT	IQ	Age^a^	Arousal (2‐1)	Arousal (3‐1)	‐
Model 16	INT	AFF	LIF	ANT	IQ	Age^a^	Arousal (2‐1)	Arousal (3‐2)	‐
Model 17	INT	AFF	LIF	ANT	IQ	Age^a^	Arousal (3‐2)	Arousal (3‐1)	‐

Abbreviations: IVs, Independent variables; PCL‐R, Psychopathy Checklist‐Revised.

^a^ Paths between the age variable and PCL‐R INT, PCL‐R LIF, and PCL‐R ANT were trimmed given non‐significance.

To test our hypotheses of distinct decision outcomes based on spontaneous or deliberate emotion processing, we used difference score criterion variables between task conditions. Thus, we ran separate models including the task condition difference scores (2‐1), (3‐2), and (3‐1) as dependent variables (see path models 2–4 for moral judgment, and models 6–8 for choice of action differences). With regard to the hypothesized change in moral decisions based on deliberate emotion processing, differences in decisions between the deliberate (3) and the spontaneous (2) were of particular interest.

We pursued the same procedure for valence and arousal ratings, by mapping the distinct ratings per condition onto the separate facets (models 10 and 14) and included the differences between valence (models 11–13) and arousal (models 15–17) ratings into the models. Total moral judgment ratings and choices of action were included in supplemental analyses, to test the hypothesized distinction between judging and choosing (model 9).

Exact model fit was assessed using the *χ* ^2^ test. If the *χ* ^2^ test is not significant (i.e., *p* > 0.05), it is assumed that the difference between the reproduced covariance matrix of the model and the observed covariance matrix of the sample is not significantly different from zero and thus, that the hypothesized model accounts for the sample data sufficiently well. The Comparative Fit Index (CFI) and the Tucker Lewis Index (TLI) are reported for incremental fit, with values of 0.95 or above signifying good model fit. Also, absolute fit was evaluated with the Standardized Root Mean Square Residual (SRMR) and the Root Mean Square Error of Approximation (RMSEA) to gauge how well the path models accounted for the data. This is assumed with a recommended value of ≤0.08 for the SRMR, and ≤0.05 for the RMSEA. Path modeling analyses were conducted with Mplus 7.0 (Muthén & Muthén, 2010).

## 4 RESULTS

As expected, bivariate correlations revealed no significant relation between the PCL‐R total score and the moral dilemma outcome variables, but distinct associations between the four psychopathy facets and the dependent variables (see Tables 2 and 3). Significant negative correlations between the Affective facet and moral judgment and the choice of action in the spontaneous emotion processing condition indicated that high Affective facet scores were related to less utilitarian judgments in the presence of a spontaneous emotion feedback. The Affective facet was significantly linked to a more pleasant experience in the spontaneous emotion feedback condition compared to the baseline (2‐1). The same association was found for the Interpersonal facet. The Antisocial facet was linked to more reported excitement during decisions in the deliberate emotion processing condition compared to the spontaneous emotion processing condition (3‐2). IQ was negatively associated with utilitarian choice of action decisions in the deliberate emotion processing condition, and also with the difference scores comparing the deliberate to the spontaneous (3‐2) and to the baseline condition (3‐1). Furthermore, age was related to significantly more unpleasant experiences during decisions in the baseline.

**TABLE 2:** Bivariate correlations among PCL‐R facets, covariates, and moral decision outcome variables; *N* = 60

Variables	1	2	3	4	5	6	7	8	9	10	11	12	13	14	15	16	17	18
1. PCL total	(0.87)	‐	‐	‐	‐	‐	‐	‐	‐	‐	‐	‐	‐	‐	‐	‐	‐	‐
2. PCL INT	0.74**	(0.81)	‐	‐	‐	‐	‐	‐	‐	‐	‐	‐	‐	‐	‐	‐	‐	‐
3. PCL AFF	0.77**	0.64**	(0.85)	‐	‐	‐	‐	‐	‐	‐	‐	‐	‐	‐	‐	‐	‐	‐
4. PCL LIF	0.82**	0.43**	0.47**	(0.78)	‐	‐	‐	‐	‐	‐	‐	‐	‐	‐	‐	‐	‐	‐
5. PCL ANT	0.78*	0.30**	0.44**	0.66**	(0.76)	‐	‐	‐	‐	‐	‐	‐	‐	‐	‐	‐	‐	‐
6. Age	0.14	0.17	0.27*	−0.02	−0.02	‐	‐	‐	‐	‐	‐	‐	‐	‐	‐	‐	‐	‐
7. IQ	−0.16	−0.18	−0.13	−0.04	−0.17	−0.23*	‐	‐	‐	‐	‐	‐	‐	‐	‐	‐	‐	‐
8. Judgment 1	−0.16	−0.04	−0.20	−0.20	−0.11	0.06	0.04	(0.73)	‐	‐	‐	‐	‐	‐	‐	‐	‐	‐
9. Judgment 2	−0.11	−0.06	−0.28*	−0.09	0.04	0.04	0.04	0.81**	(0.84)	‐	‐	‐	‐	‐	‐	‐	‐	‐
10. Judgment 3	−0.08	−0.03	−0.20	−0.05	0.00	0.05	−0.07	0.63**	0.81**	(0.84)	‐	‐	‐	‐	‐	‐	‐	‐
11. Judgment 2‐1	0.04	−0.06	−0.12	0.15	0.19	−0.00	0.02	−0.20	0.41**	0.31**	‐	‐	‐	‐	‐	‐	‐	‐
12. Judgment 3‐2	0.02	0.04	0.14	0.06	−0.10	0.05	−0.19	−0.24*	−0.32**	0.30*	−0.17	‐	‐	‐	‐	‐	‐	‐
13. Judgment 3‐1	0.08	0.01	−0.01	0.17	0.13	0.00	−0.13	−0.38**	0.06	0.49**	0.63**	0.66**	‐	‐	‐	‐	‐	‐
14.Choice 1	−0.08	0.02	−0.13	−0.12	−0.06	0.16	−0.03	0.89**	0.73**	0.59**	−0.16	−0.17	−0.30**	(0.73)	‐	‐	‐	‐
15.Choice 2	−0.10	0.00	−0.22*	−0.13	0.02	0.01	−0.07	0.67**	0.76**	0.69**	0.24*	−0.12	0.09	0.73**	(0.79)	‐	‐	‐
16.Choice 3	0.01	0.05	−0.14	−0.03	0.10	0.04	−0.22*	0.53**	0.63**	0.85**	0.16	0.33**	0.41**	0.55**	0.81**	(0.88)	‐	‐
17. Choice 2‐1	−0.05	−0.05	−0.10	−0.01	0.06	−0.19	−0.05	−0.31**	0.04	0.08	0.55**	0.07	0.47**	−0.38**	0.36**	0.28*	‐	‐
18. Choice 3‐2	0.13	0.06	0.11	0.13	0.09	0.07	−0.27*	−0.02	−0.07	0.39**	−0.08	0.73**	0.52**	−0.07	−0.12	0.49**	−0.07	‐
19. Choice 3‐1	0.08	0.04	−0.03	0.09	0.16	−0.12	−0.21*	−0.29*	−0.02	0.36**	0.36**	0.57**	0.76**	−0.37**	0.19	0.57**	0.72**	0.65**

*Note*: Reliability estimates in brackets are Cronbach's alpha values for internal consistency. Bold values are significant correlations between IVs and DVs. Experimental conditions and difference scores between the conditions are indicated by numbers (1 = baseline, 2 = spontaneous emotion processing, and 3 = deliberate emotion processing).

Abbreviations: DVs, dependent variables; PCL‐R, Psychopathy Checklist‐Revised.

*p < 0.05, **p < 0.01.

**TABLE 3:** Bivariate correlations between PCL‐R facets, covariates, and valence and arousal; *N* = 60

Variables	1	2	3	4	5	6	7	8	9	10	11	12	13	14	15	16	17	18
1. PCL total	(0.87)	‐	‐	‐	‐	‐	‐	‐	‐	‐	‐	‐	‐	‐	‐	‐	‐	‐
2. PCL INT	0.74**	(0.81)	‐	‐	‐	‐	‐	‐	‐	‐	‐	‐	‐	‐	‐	‐	‐	‐
3. PCL AFF	0.77**	0.64**	(0.85)	‐	‐	‐	‐	‐	‐	‐	‐	‐	‐	‐	‐	‐	‐	‐
4. PCL LIF	0.82**	0.43**	0.47**	(0.78)	‐	‐	‐	‐	‐	‐	‐	‐	‐	‐	‐	‐	‐	‐
5. PCL ANT	0.78^a^	0.30**	0.44**	0.66**	(0.76)	‐	‐	‐	‐	‐	‐	‐	‐	‐	‐	‐	‐	‐
6. Age	0.14	0.17	0.27^a^	−0.02	−0.02	‐	‐	‐	‐	‐	‐	‐	‐	‐	‐	‐	‐	‐
7. IQ	−0.16	−0.18	−0.13	−0.04	−0.17	−0.23^a^	‐	‐	‐	‐	‐	‐	‐	‐	‐	‐	‐	‐
8. Valence 1	−0.01	−0.03	−0.01	−0.02	−0.01	−0.22^a^	0.04	(0.64)	‐	‐	‐	‐	‐	‐	‐	‐	‐	‐
9. Valence 2	0.12	0.18	0.22	−0.07	0.05	−0.04	0.03	0.69**	(0.80)	‐	‐	‐	‐	‐	‐	‐	‐	‐
10. Valence 3	0.06	0.07	0.16	−0.03	0.01	−0.10	0.09	0.64**	0.77**	(0.88)	‐	‐	‐	‐	‐	‐	‐	‐
11. Valence 2‐1	0.17	**0.27** ^a^	**0.26** ^a^	−0.06	0.10	0.21	−0.02	−0.37**	0.42**	0.19	‐	‐	‐	‐	‐	‐	‐	‐
12. Valence 3‐2	−0.09	−0.16	−0.03	0.04	−0.10	−0.07	0.10	0.00	−0.23^a^	0.45**	−0.30	‐	‐	‐	‐	‐	‐	‐
13. Valence 3‐1	0.08	0.11	0.20	−0.02	0.02	0.12	0.07	−0.31**	0.19	0.53**	0.64**	0.55**	‐	‐	‐	‐	‐	‐
14. Arousal 1	−0.03	0.03	−0.07	0.03	−0.02	0.05	−0.06	−0.48**	−0.43**	−0.39**	0.04	0.03	0.05	(0.78)	‐	‐	‐	‐
15. Arousal 2	−0.12	−0.07	−0.20	0.05	−0.14	0.01	−0.02	−0.54**	−0.58**	−0.46**	−0.06	0.10	0.03	0.61**	(0.83)	‐	‐	‐
16. Arousal 3	0.01	0.01	−0.12	0.12	0.02	−0.02	−0.09	−0.60**	−0.58**	−0.60**	−0.00	−0.09	−0.08	0.65**	0.79**	(0.87)	‐	‐
17. Arousal 2‐1	−0.15	−0.15	−0.14	0.01	−0.21	−0.03	0.05	−0.09	−0.18	−0.11	−0.16	0.08	−0.03	−0.42**	0.46**	0.19	‐	‐
18. Arousal 3‐2	0.22	0.13	0.10	0.12	0.28*	−0.06	−0.11	−0.07	0.01	−0.19	0.09	−0.30^a^	−0.16	0.03	−0.34**	0.31**	−0.42**	‐
19. Arousal 3‐1	0.05	−0.03	−0.07	0.12	0.05	−0.08	−0.04	−0.13	−0.18	−0.24*	−0.05	−0.15	−0.15	−0.43**	0.22^a^	0.41**	0.71**	0.33**

*Note*: Reliability estimates in brackets are Cronbach's alpha values for internal scale consistency. Bold values are significant correlations between IV's and DVs. Experimental conditions and difference scores between the conditions are indicated by numbers (1 = baseline, 2 = spontaneous emotion processing, and 3 = deliberate emotion processing).

Abbreviations: DVs, dependent variables; PCL‐R, Psychopathy Checklist‐Revised.

*p < 0.05. **p < 0.01.

### 4.1 Path analyses

#### 4.1.1 Moral judgment and choice of action

**FIGURE 2:**
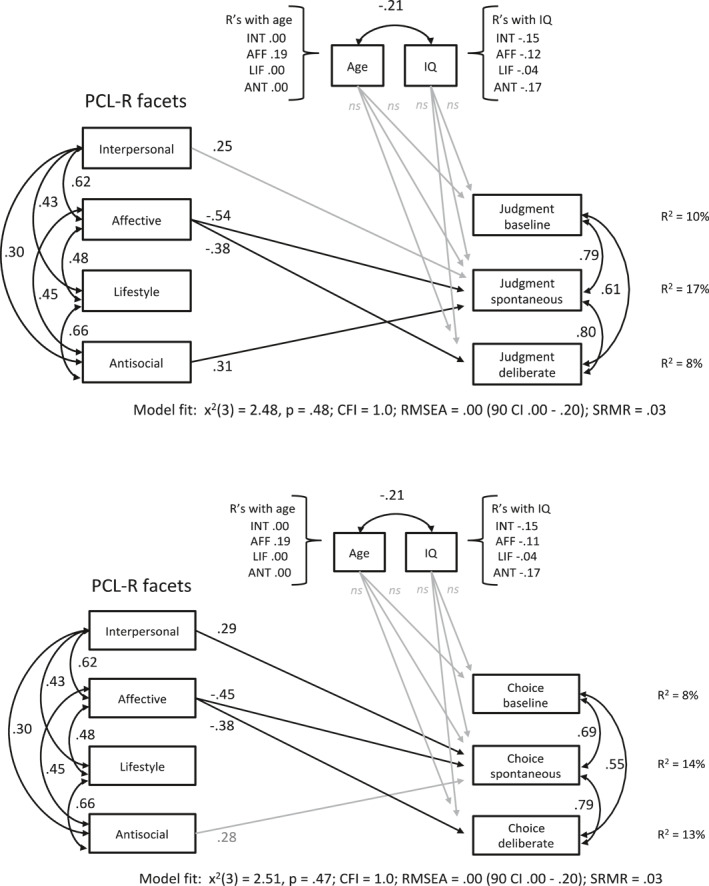
Path models 1 and 5 with Psychopathy Checklist‐Revised (PCL‐R) facets predicting judgments (upper model) and choices (lower model) in separate conditions. +Significant predictors are illustrated as black arrows, gray arrows for dependent variables indicate predictions at trend level. Age and IQ are included as covariates. CFI, Comparative Fit Index; RMSEA, Root Mean Square Error of Approximation; SRMR, Standardized Root Mean Square Residual

Utilitarian decisions in the three experimental conditions were analyzed in separate path models for moral judgment (model 1; Figure 2) and choice of action outcomes (model 5; Figure 2) and regressed on the four PCL‐R facets. Non‐significant paths were trimmed as indicated in Table 1 (see also Carré et al., 2013, for this procedure in earlier research). The models fit the data well (for fit indices of the separate decision outcome models, see Table 4). The Affective facet was a strong predictor for deontological/non‐utilitarian moral judgments (*β* = −0.54, *SE* = 0.14, *p* = 0.000 [90% CI = −0.77, −0.30]) in model 1 and choice of action ratings in model 5 (*β* = −0.45, *SE* = 0.12, *p* = 0.001 [90% CI = −0.68, −0.22]) in the spontaneous emotion feedback condition.

**TABLE 4:** Path analyses results: Model fit indices for separate models with moral dilemma DVs moral judgment, choice of action, and corresponding difference scores

Model and DVs		*χ* ^2^ (*df*)	*p*‐value	CFI	TLI	RMSEA (90% CI)	SRMR	Variance explained (*R* ^2^)
1. Moral judgment	1	2.477 (3)	0.479	1.000	1.033	0.000	0.027	0.09
	2					(0.000, 0.203)		0.17*
	3							0.08
2. Difference MJ	2‐1	2.478 (3)	0.479	1.000	1.078	0.000	0.030	0.06
	3‐1					(0.000, 0.203)		0.14
3. Difference MJ	2‐1	2.477 (3)	0.479	1.000	1.694	0.000	0.030	0.11
	3‐2					(0.000, 0.203)		0.16
4. Difference MJ	3‐2	2.478 (3)	0.479	1.000	1.064	0.000	0.029	0.10
	3‐1					(0.000, 0.203)		0.06
5. Choice of action	1	2.506 (3)	0.474	1.000	1.042	0.000	0.027	0.08
	2					(0.000, 0.203)		0.14
	3							0.13
6. Difference COA	2‐1	2.482 (3)	0.479	1.000	1.065	0.000	0.030	0.09
	3‐1					(0.000, 0.203)		0.10
7. Difference COA	2‐1	2.477 (3)	0.479	1.000	1.000	0.000	0.030	0.06
	3‐2					(0.000, 0.203)		0.10
8. Difference COA	3‐2	2.482 (3)	0.0479	1.000	1.083	0.000	0.030	0.09
	3‐1					(0.000, 0.203)		0.10
9. Choices & judgments	Total ratings	2.497 (3)	0.0476	1.000	1.03	0.000	0.030	0.14*
						(0.000, 0.203)		0.14

*Note*: DVs: Numbers represent task conditions with 1 = baseline, 2 = spontaneous, and 3 = deliberate emotion condition.

Abbreviations: CFI, Comparative Fit Index; DVs, dependent variables; PCL‐R, Psychopathy Checklist‐Revised; RMSEA, Root Mean Square Error of Approximation; SRMR, Standardized Root Mean Square Residual; TLI, Tucker Lewis Index.

*p < 0.05.

The Affective facet also predicted deontological moral judgments in the deliberate condition (*β* = −0.38, *SE* = 0.15, *p* = 0.013 [90% CI = −0.63, −0.13]) and comparably, choices of action (*β* = −0.38, *SE* = 0.14, *p* = 0.017 [90% CI = −0.64, −0.12]). The Antisocial facet on the other hand predicted more acceptance ratings of utilitarian judgments in the presence of spontaneous emotion feedback (*β* = 0.31, *SE* = 0.14, *p* = 0.025 [90% CI = 0.08, 0.54]) as well as the willingness to engage in such behavior (*β* = 0.28, *SE* = 0.13, *p* = 0.063 [90% CI = 0.03, 0.54]). The Interpersonal facet predicted utilitarian moral judgments at trend level (*β* = 0.25, *SE* = 0.14, *p* = 0.078 [90% CI = 0.02, 0.49]) and choices of action significantly (*β* = 0.29, *SE* = 0.11, *p* = 0.038 [90% CI = 0.06, 0.53]) in the spontaneous emotion processing condition. Results of total moral judgments and choices taken together across conditions (model 9) revealed a significant prediction of the Interpersonal facet for more utilitarian choices (*β* = 0.29, *SE* = 0.15, *p* = 0.048 [90% CI = 0.05, 0.53]) but not judgments (*β* = 0.25, *SE* = 0.14, *p* = 0.077 [90% CI = 0.02, 0.49]). The Affective facet was highly predictive for less utilitarian decisions in both judgments (*β* = −0.48, *SE* = 0.15, *p* = 0.001 [90% CI = −0.72, −0.24]) and choices (*β* = −0.44, *SE* = 0.15, *p* = 0.003 [90% CI = ‐ 0.69, −0.19]). The Antisocial facet was associated with utilitarian responses at trend level (judgments: *β* = 0.23, *SE* = 0.15, *p* = 0.108 [90% CI = −0.01, 0.47]; choices: *β* = 0.26, *SE* = 0.15, *p* = 0.081 [90% CI = 0.02, 0.51]).

#### 4.1.2 Spontaneous and deliberate emotion processing in moral decisions

**FIGURE 3:**
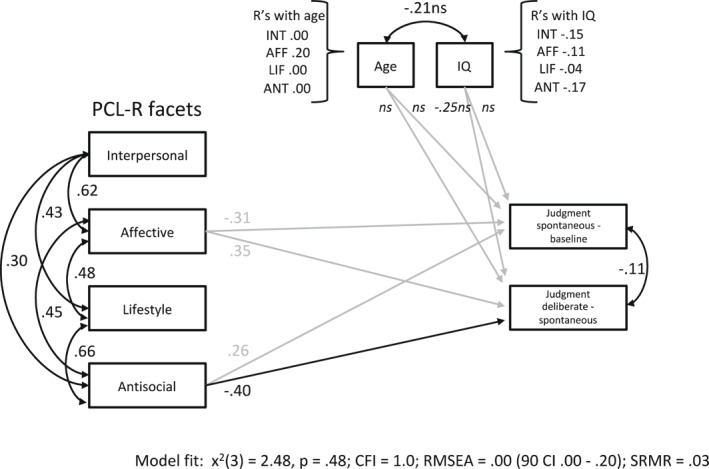
Path model 3 with Psychopathy Checklist‐Revised (PCL‐R) facets predicting moral judgment difference scores. Significant predictors are illustrated as black arrows, gray arrows for dependent variables indicate predictions at trend level. Age and IQ are included as covariates. CFI, Comparative Fit Index; RMSEA, Root Mean Square Error of Approximation; SRMR, Standardized Root Mean Square Residual

The inclusion of difference scores between the separate task conditions along with age and IQ as covariates in model 3 (see Figure 3) revealed a significant prediction of the Antisocial facet for less utilitarian moral judgments in the deliberate (3) compared to the spontaneous (2) emotion feedback condition (*β* = −0.40, *SE* = 0.18, *p* = 0.022 [90% CI = −0.69, −0.11]). The Affective facet predicted more utilitarian judgments in the deliberate (3) compared to the spontaneous (2) condition at trend level (*β* = 0.35, *SE* = 0.20, *p* = 0.078 [90% CI = 0.02, 0.67]). Also at trend level, higher IQ scores were associated with less utilitarian judgments between these (3‐2) conditions (*β* = −0.25, *SE* = 0.14, *p* = 0.070 [90% CI = −0.48, −0.02]). Difference scores in choice of action were not, however, significantly related to the PCL‐R facets, age, or IQ.

#### 4.1.3 Valence and arousal in moral decisions

In additional path models, we further explored how pleasant the decision‐making was experienced via a valence rating and how excited participants felt during their choices via arousal rating. The higher the valence score, the more pleasant the decision‐making was experienced and the higher the arousal score, the more excited participants reportedly felt.

**FIGURE 4:**
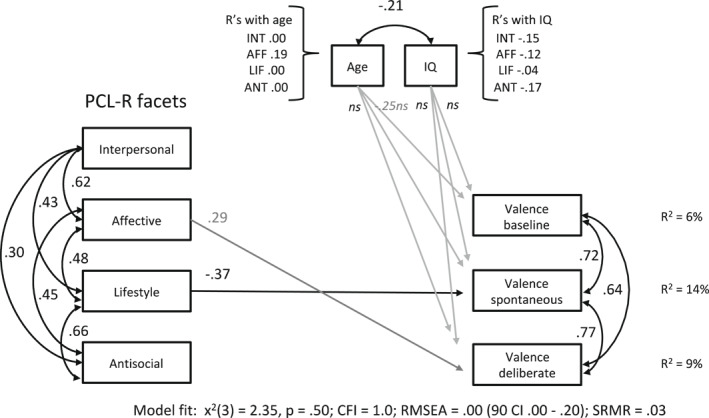
Path model 10 with Psychopathy Checklist‐Revised (PCL‐R) facets predicting valence ratings in separate conditions. Significant predictors are illustrated as black arrows, gray arrows for dependent variables indicate predictions at trend level. Age and IQ are included as covariates. CFI, Comparative Fit Index; RMSEA, Root Mean Square Error of Approximation; SRMR, Standardized Root Mean Square Residual

**FIGURE 5:**
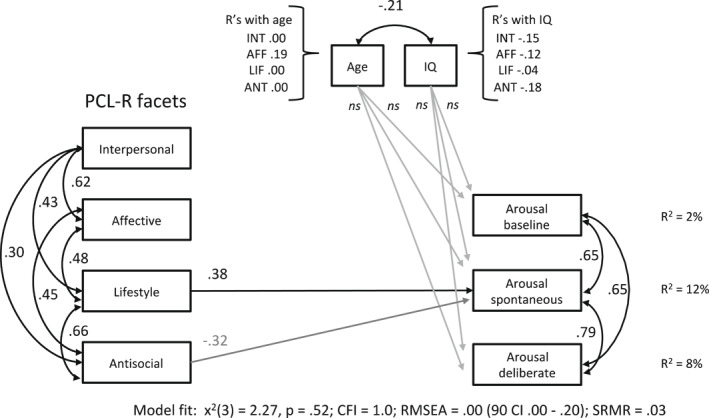
Path model 14 with Psychopathy Checklist‐Revised (PCL‐R) facets predicting arousal ratings in separate conditions. Significant predictors are illustrated as black arrows, gray arrows for dependent variables indicate predictions at trend level. Age and IQ are included as covariates. CFI, Comparative Fit Index; RMSEA, Root Mean Square Error of Approximation; SRMR, Standardized Root Mean Square Residual

The models fit the data well (see fit indices in Figures 4 and 5). The valence rating in the baseline condition was negatively associated with age (*β* = −0.25, *SE* = 0.13, *p* = 0.053 [90% CI = −0.47, −0.04]) in model 10, indicating that higher age led to a more unpleasant experience in moral decisions. Age was unrelated to arousal ratings, however, in all conditions (model 14). The Lifestyle facet predicted an unpleasant experience (valence) and excitement (arousal) during decisions in the spontaneous condition only (valence: *β* = −0.37, *SE* = 0.18, *p* = 0.041 [90% CI = −0.67, −0.07]; arousal: *β* = 0.38, *SE* = 0.19, *p* = 0.042 [90% CI = 0.07, 0.68]). The Affective facet predicted pleasantly experienced decision‐making in the deliberate emotion processing condition at trend level (*β* = 0.29, *SE* = 0.17, *p* = 0.086 [90% CI = 0.01, 0.29]) but did not reveal a significant relation to reported excitement during decisions.

#### 4.1.4 Valence and arousal in spontaneous versus deliberate emotion processing

When including difference scores into the models, the Lifestyle facet predicted significantly less pleasant decision‐making (*β* = −0.38, *SE* = 0.15, *p* = 0.008 [90% CI = −0.62, −0.14]) and more excitement (*β* = 0.41, *SE* = 0.17, *p* = 0.015 [90% CI = 0.13, 0.69]) experienced with spontaneous emotion feedback compared to the baseline condition (2‐1). The Antisocial facet predicted in the other direction with a more pleasant experience at trend level (*β* = 0.24, *SE* = 0.13, *p* = 0.063 [90% CI = 0.03, 0.44]) and highly significant less excitement (*β* = −0.44, *SE* = 0.15, *p* = 0.004 [90% CI = −0.69, −0.19]). The Interpersonal facet was negatively related to the difference score (3‐2) for valence (*β* = −0.30, *SE* = 0.16, *p* = 0.055 [90% CI = −0.56, −0.04]) but unrelated to arousal, indicating that higher Interpersonal facet scores predicted a more unpleasant decision‐making process when deliberately processing emotions of the proposed victims compared to a spontaneous emotion processing with no difference in excitement. The Affective facet was related to the opposite trend, with higher scores predicting a more pleasant decision‐making process with the deliberate compared to no emotion feedback (*β* = 0.23, *SE* = 0.14, *p* = 0.092 [90% CI = 0.01, 0.46], see model 13), but no relation was found between the Affective facet and arousal difference scores (3‐1). The Lifestyle facet though, was associated with more reported excitement during decisions in the deliberate emotion processing condition compared to the baseline (*β* = 0.30, *SE* = 0.18, *p* = 0.093 [90% CI = 0.01, 0.58]), but was unrelated to how pleasant the decision‐making was experienced.

## 5 DISCUSSION

In this study, we investigated whether criminal offenders with varying levels of psychopathic traits show differences in moral decision‐making processes according to the focus on emotion feedback received after their decisions. Previous research has found more utilitarian moral decision patterns among individuals elevated on psychopathic traits, whereas other studies were not able to corroborate this finding (see Marshall et al., 2018, for an overview). When examining psychopathy in terms of a unitary construct (using PCL‐R total score), no meaningful associations with moral decisions emerged for the present sample. With the use of path models to simultaneously map the separate psychopathy facets onto distinct decision outcomes, however, we found differential associations between aspects of moral decisions and the four psychopathic domains, signifying the importance of recognizing the multifarious nature of psychopathic personality (Neumann et al., 2015).

### 5.1 Judging and choosing

Based on studies claiming that the differentiation between judging morally and choosing to act upon these judgments plays an important role in the moral compass of psychopathic individuals (Cima et al., 2010; Pletti et al., 2017; Tassy et al., 2013), we included this distinction in the current study. We expected psychopathy to be predictive for utilitarian choice of actions but not for utilitarian moral judgment. In line with this, we did not find a significant relationship between the psychopathy facets and utilitarian moral judgments (without emotion feedback; baseline condition in model 1). But, unlike previous research, we also did not find psychopathic traits to predict utilitarian moral actions either (baseline condition in model 5).

In an additional path model, we analyzed the total moral judgments and choices taken together across conditions.^3^ Results indicate that the Interpersonal facet predicted more utilitarian choices but not judgments (“acceptable or not, I'd do it”). The Affective facet was highly predictive for less utilitarian choices in both judgments (“it's inappropriate”) and choices (“I wouldn't do it”). These results magnify the trend that we found for the associations between the Interpersonal facet and the baseline decision outcomes and between the Affective facet and the baseline decision outcomes. Our findings are at odds, however, with the suggested disparity between moral judgments and choices of action in psychopathic individuals (e.g., Pletti et al., 2017; Tassy et al., 2013). Instead, our findings suggest that the Interpersonal PCL‐R facet accounts for the willingness to act upon utilitarian guidelines without a link to a theoretical preference in moral judgments. This finding supports the view that some psychopathic traits are related to an indifference to moral guidelines that is separable from eventual endorsement of such behavior (see cf. Cima et al., 2010; Luke et al., 2021). In line with this, earlier research has linked utilitarian choices with impaired social cognition rather than with deliberative reasoning (Duke & Bègue, 2015).

### 5.2 Spontaneous and deliberate emotion processing alter moral decisions

Theoretical sacrificial moral dilemmas have been a subject of debate about their qualification to measure empathic correlates (Marshall et al., 2018). Some argue that the far‐fetched dilemmas already require a large effort of mentalizing *per se* and thus exacerbate theory of mind deficits in psychopathic individuals (Bostyn et al., 2019). The lack of empathy in psychopathy has recently been associated with a deficit in psychopathic individuals to automatically empathize with others, despite an intact ability to deliberately take the perspective of another person (Drayton et al., 2018). As indicated by our results, it remains an open question whether and in which direction the deliberate processing of other's emotions in moral dilemmas influence decision‐making processes in psychopathic individuals.

In our study, the distinct processing of emotional content in moral dilemmas (spontaneous vs. deliberate) was linked to divergent associations with PCL‐R facets. Also, individual psychopathic traits were only related to utilitarian decisions (i.e., Interpersonal and Antisocial facet) and deontological decisions (i.e., the Affective facet) with the additional presence of emotion feedback. Earlier findings have suggested that psychopathic offenders compared with non‐psychopathic offenders draw on cognitive compensation strategies when confronted with moral tasks, which helps them to process emotional information despite neural abnormalities (Glenn et al., 2009; Kiehl et al., 2001; Yoder et al., 2015). Specifically, this compensation would be mirrored in altered brain activity during moral decisions despite comparable behavioral choices to individuals presenting with low psychopathic traits (Glenn et al., 2009). It has been largely shown, however, that these compensation strategies that supposedly inflate cognitive empathy in psychopathy are strongly influenced by adding to the cognitive load (Baskin‐Sommers et al., 2013; Meffert et al., 2013; Vieira et al., 2014). In our study, the addition of emotional information led to altered moral judgments on a behavioral level. Thus, the additional emotional information might have interfered with said cognitive compensation strategies by enhancing the cognitive load, offering further support to this view.

As an underlying mechanism influencing more utilitarian decisions in psychopathic individuals, a reduced emotional response to the harming of others has been discussed (Blair, 2007; Pletti et al., 2017). Without emotion feedback, our findings did not confirm this view according to subjective evaluations in our offender sample. Nevertheless, the emotion feedback changed the reported experience on a multi‐faceted level. Along with deontological judgments predicted by the Affective facet, these callous traits were predictive of a pleasant experience when deliberately processing a victim's emotions. This finding strengthens earlier research reporting an association between affective psychopathic features with enjoying harmful interactions (Yoder et al., 2015).

Conversely, the Antisocial facet was negatively related to arousal during pronounced utilitarian judgments in the spontaneous emotion processing condition, which was especially emphasized in the contrast to the dilemmas without emotion feedback. As distinguished from the Antisocial facet, the Lifestyle facet was predictive of an unpleasant experience with a positive relation to excitement. This is noteworthy since the different psychopathy facets were not only associated with varying decisions in the moral dilemmas but also the subjective experience largely diverged. Against this backdrop, further studies should consider the distinct psychopathic traits as multi‐faceted predictors in the moral decision finding process.

Moreover, our results revealed that the deliberate processing of an imagined victim's emotion led to more utilitarian judgments related to the Affective facet and to less utilitarian judgments related to the Antisocial facet compared with a spontaneous emotion feedback. These results support the hypothesis of distinct deliberate and spontaneous emotion processing in psychopathy, consistent with new research suggesting psychopathic individuals can up‐regulate neuro‐affective processes when asked to do so (Drayton et al., 2018; Shane & Groat, 2018). At the behavioral level, however, the deliberate processing of the emotions of others and, thus, the presumed leveling of the emotion processing deficit in psychopathic individuals does not automatically lead to a more prosocial decision style in these individuals. While earlier research suggests that more emotional empathy leads to more prosocial decisions (Blair, 1995), our study challenges this view. Our argument for a more differentiated concept of the empathic deficit in psychopathy is in line with research reporting that only about 1% of the variation in aggression is accounted for by empathy, demarcating moral conduct from empathy (Vachon et al., 2014). Similarly, a recent study found aggression to mediate the relationship between sub‐clinical psychopathy and utilitarian decisions (Balash & Falkenbach, 2018). On a related note, moral philosophers and psychologists have questioned whether empathy is an essential precondition for morality. Even more, they made the point that empathy, in contrast to attention and concern, does not matter for moral regard (Maibom, 2009; Prinz, 2011). Thus, empathy has been considered an even harmful role to moral decisions due to its subjective and biasing motivational character that disables a fair and compassionate judgment (Bloom, 2016; Breithaupt, 2019).

### 5.3 Are utilitarian decisions rational and deontological decisions emotional?

Using path models along with age and IQ as covariates, the four PCL‐R facets as predictors and the separate task conditions as outcome variables revealed that the Affective facet of psychopathy predicted non‐utilitarian judgments in sacrificial moral dilemmas in the presence of emotion feedback. One explanation for this result is that non‐utilitarian judgments and choices do not imply just intuitive, emotional concern as claimed by several researchers (Greene et al., 2001, 2004; Singer, 2005; Takamatsu & Takai, 2019; Unger, 1996). Beyond that, in a pathological dimension, cold‐heartedness and a lack of empathy could be a predictor for deontological decisions not for the sake of deeper underlying moral principles but because of a profound carelessness and unwillingness to engage in moral behavior of any kind. This is because utilitarian decisions are traditionally coded positively (questions like “would you sacrifice one for many?” are coded insofar that the more approval, the more utilitarian is the response pattern). This could imply that deontological moral decisions are possibly chosen not only for discussed reasons of virtue and duty, but also to elude or defy from moral decisions by denial. As predicted by extreme manifestations of unempathic traits, an alternative cognitive route to strong deontological decisions could thus be found in an attitude of keeping one's hands clean and staying out of any social involvement, whatsoever. In line with this interpretation, consistent deontological responses in sacrificial and real world dilemmas were recently linked to “morally unconcerned” persons who presented with significantly lower empathic concern (Rosas et al., 2019, p. 131).

Relatedly, the deontological decisions coupled with a pleasant experience linked to the affective (more appropriately: affectionless) psychopathy trait suggests that low empathy is linked to enjoyment in hurting more people, respectively letting more people die. Therefore, beyond a lack of concern that implies indifference toward the harm of others, the reported pleasantness during deontological decisions associated with low empathic traits implies joy in experiencing the harm of others. Recent research has shown that there is indeed a psychopathic domain that pertains to enjoyment in doing harm (Roy, Neumann, et al., 2021; Roy, Vize, et al., 2021). In this sense, deontological decisions might indicate absent harm aversion that favors the death of more people. This is in contrast to the notion that harm aversion hinders individuals from making utilitarian decisions due to the active involvement in sacrificing one person for many (Gleichgerrcht & Young, 2013). Interestingly, in a factor‐analytic study of the PCL‐R and a measure of severe sexual sadism within a sample of forensic‐psychiatric patients including 50 diagnosed with Sexual Sadism Disorder, the two constructs of psychopathy and sexual sadism could clearly be distinguished; but a single item from the PCL‐R Affective facet was closer to the sadism factor than to the psychopathy factor (Mokros et al., 2011). Moreover, psychopathy shows strong associations with non‐sexual sadism (i.e., everyday sadism; Paulhus & Dutton, 2016). Further research is needed to investigate this assumption by adding methodological variation to moral dilemma studies.

Interestingly, in our study, intelligence was related to more deontological judgments in the last (i.e., deliberate) compared to the second condition (i.e., spontaneous). This finding is in contrast to earlier studies that reported deliberate thinking patterns to be associated with more utilitarian judgments, which was linked to higher working memory capacities (Moore et al., 2008). It is plausible that the additional deliberate emotion processing in the task added to the cognitive load in the last condition. In light of earlier research, however, it is surprising that higher IQ scores were related to more deontological decisions in the context of this additional task. This result raises doubt as to whether deontological judgments should be understood as intuitive heuristics. Inversely, it questions whether utilitarian judgments represent the more rational moral reflection. It is conceivable that the integration of emotions in decision‐making processes leads to more deontological decisions and that the additional emotional information was more absorbable in case of higher cognitive capacities. Our findings suggest that the integration of emotional information into decision‐making processes should not be understood as a cognitive error in the sense of heuristics. Rather, it might reflect a cognitively demanding and necessary process that underlies the complex route to moral decision‐making.

### 5.4 Limitations

Although we controlled for mean differences in conflict ratings of moral dilemmas between the separate conditions in our pilot study sample, the comparison of distinct dilemmas in the three conditions is a possible confounding factor for attributing the within‐subject differences to the emotion interference. Moreover, moral dilemmas were not randomized among experimental conditions to avoid randomization as confounding factor (Saint‐Mont, 2015). However, this complicates the exact comparability among dilemmas. Future research using moral dilemmas in experimental set‐ups that include different conditions should take this issue into account and consider randomizing the dilemmas among conditions (Luke et al., 2021).

The implementation of spontaneous versus deliberate emotion processing in imagined, theoretical sacrificial moral dilemma scenarios remain delicate by the very nature of these tasks. For obvious ethical reasons, it is unthinkable to involve real victims in a task that would allow the participants to cause suffering in order to manipulate the deliberation of the perpetuators' emotional responsiveness. One recent approach of real‐life dilemmas was conducted with monetary rewards (Bostyn et al., 2019). The authors of this study used monetary dilemmas, though, which is hardly comparable to the sacrificing of lives. Although the classical sacrificial dilemmas might be far‐fetched, the stretch to monetary dilemmas is a qualitative difference that in consequence leads to utterly different conclusions when comparing psychopathic criminal offenders characterized by callousness from healthy individuals with intact emotion regulation. We solved this problem with the proposed differentiation between men and women (spontaneous condition) versus between separate emotions (deliberate condition). It is conceivable though, that the introduction of gender as the focus on the imagined victim played a role that influenced the empathic reaction of participants. We controlled for this issue by evenly balancing out male and female faces in the deliberate as well as in the spontaneous condition. Still, it remains possible, that psychopathic offenders depending on their crimes (e.g., sexual or violent offenders) vary strongly in their empathic response toward female versus male victims, be it of their real life or imagined conduct.

## 6 IMPLICATIONS AND CONCLUSION

Studies on psychopathy and morality using sacrificial dilemmas have resulted in heterogeneous findings. While some researchers have claimed utilitarian judgment to be characteristic for psychopathic individuals, others found no such relation. In our study, the four psychopathy facets of the PCL‐R revealed contrasting associations to moral decisions. It therefore adds to the current knowledge by reuniting the formerly contradictory findings and suggests that the heterogeneity of other studies is related to the multi‐faceted structure of the psychopathy construct. Indeed, several studies reported distinct associations of the psychopathy facets to moral correlates (Gao & Tang, 2013; Glenn et al., 2009; Schaich Borg et al., 2013). Increasing evidence shows that even if psychopathy can be understood as a cohesive syndrome constituted of subdimensions, these symptom subdimensions are predictive of different correlates (Garofalo et al., 2019; Hare & Neumann, 2008; Neumann & Hare, 2008; Patrick, 2018). This finding should be considered in future research investigating cognitive‐behavioral and moral correlates of psychopathy.

Moreover, in this study, we paid tribute to the growing evidence referring to the dimensional character of the psychopathic construct with a path analytic approach (Patrick, 2018). Our modeling results support the view of a multi‐dimensional psychopathy construct. Nevertheless, in spite of our affirmation to treat the psychopathy construct along a continuum, we stress the importance of further moral investigations in offender samples. To date, most research exploring moral peculiarities in psychopathy has been conducted with college samples. In previous studies, utilitarian choices were mostly correlated with subclinical psychopathy with limited applicability to psychopathy in clinical terms (with the exception of Glenn et al., 2010; Koenigs et al., 2012, but for contradicting findings see Cima et al., 2010; Glenn et al., 2009; for a meta‐analytic overview see Marshall et al., 2018). Even though, along the psychopathy continuum, these samples can provide some indication on moral beliefs and behavior in relation to psychopathic tendencies, offender samples are substantially and by definition distinct from college samples with regard to moral (mis‐)behavior.

To our knowledge, our study is the first that included the differentiation between spontaneous and deliberate emotion processing of psychopathic individuals in the moral context of social decisions. Our findings encourage further research to take notice of these distinct processes to the better understanding of emotional, moral, and behavioral features of the psychopathic personality.

## Supporting information

supplementary Material 1

## ENDNOTES

^1^
As our hypotheses are based on the assumption that moral dilemmas evoke an emotional conflict, dilemmas were only used if they were conflicting for the forensic sample tested in this study (see Bühner, 2019, for test theoretical argumentation of this procedure). Results for reliability analyses supported the exclusion of low‐conflict moral scenarios, weakening the internal consistencies per condition scales. We previously controlled for the balance of perceived moral conflict of the separate dilemmas prescribed to the three experimental conditions in a pilot study on a small community sample (*n* = 12). Results from a repeated‐measures ANOVA on means of experienced conflicts per condition revealed no significant mean differences indicating the allocation of evenly difficult dilemmas across conditions (*V* = 0.21, *F*(2, 10) = 1.327, *p* > 0.05). For the complete set of moral dilemmas used in this study, see the Supporting Information S1.

^2^
Note that initially, we preregistered extreme group comparisons on a total score level. We departed from this approach for several reasons: First of all to account for the psychopathy construct as continuous construct, which was reflected by the sample used in this study. Secondly, variable distributions and results for PCL‐R total score levels were ambiguous which was supported by bivariate correlations between the key variables pointing toward a multi‐faceted approach of the psychopathy construct to analyze cognitive‐behavioral correlates. Further information and analyses are available from first author upon request.

^3^
The illustrated model can be found in the Supporting Information S1.
