# Supplementary material for: Rational, emotional, or both? Subcomponents of psychopathy predict opposing moral decisions
Source: Behav Sci Law. 2021 Oct 20;39(5):541–66. doi: 10.1002/bsl.2547 (PMC9298150; doi:10.1002/bsl.2547)
Supplement: Supplementary file 1 — supplementary Material 1 [file BSL-39-541-s001.docx]

*Supplementary Materials for*

‘Rational, emotional, or both? Subcomponents of psychopathy predict opposing moral decisions’

Supplementary Table S1 – Model Fit Indices for Arousal and Valence Path Models

|  |  | |  | |  | |  | |  | |  | |  | | |  |
| --- | --- | --- | --- | --- | --- | --- | --- | --- | --- | --- | --- | --- | --- | --- | --- | --- |
| **TABLE S1.** Path analyses results: model fit indices for the separate models with moral dilemma DV’s moral judgment, choice of action and corresponding difference scores | | | | | | | | | | | | | | | | |
| Model & DV’s | | | | χ² | | *p* | | CFI | | TLI | | RMSEA | | SRMR | Variance explained (R^2^) | |
|  |  |  |  | (df) | |  |  |  |  |  |  | (90% CI) | |  |  |  |
| 10. Arousal | | 1 | | 2.269 (3) | | .52 | | 1.000 | | 1.072 | | .000 | | .026 | .02 | |
|  |  | 2 | |  |  |  |  |  |  |  |  | (.000; .196) | |  | .12 | |
|  |  | 3 | |  |  |  |  |  |  |  |  |  |  |  | .08 | |
| 11. Difference arousal | | 2-1 | | 2.278 (3) | | .52 | | 1.000 | | 1.079 | | .000 | | .029 | .06 | |
|  |  | 3-1 | |  |  |  |  |  |  |  |  | (.000; .196) | |  | .13 | |
| 12. Difference arousal | | 2-1 | | 2.265 (3) | | .52 | | 1.000 | | 1.289 | | .000 | | .029 | .11 | |
|  |  | 3-2 | |  |  |  |  |  |  |  |  | (.000; .196) | |  | .15 | |
| 13. Difference arousal | | 3-2 | | 2.278 (3) | | .52 | | 1.000 | | 1.602 | | .000 | | .029 | .12 | |
|  |  | 3-1 | |  |  |  |  |  |  |  |  | (.000; .196) | |  | .06 | |
| 14. Valence | | 1 | | 2.346 (3) | | .50 | | 1.000 | | 1.055 | | .000 | | .028 | .06 | |
|  |  | 2 | |  |  |  |  |  |  |  |  | (.000; .198) | |  | .14 | |
|  |  | 3 | |  |  |  |  |  |  |  |  |  |  |  | .09 | |
| 15. Difference valence | | 2-1 | | 2.455 (3) | | .48 | | 1.000 | | 1.072 | | .000 | | .031 | .07 | |
|  |  | 3-1 | |  |  |  |  |  |  |  |  | (.000; .202) | |  | .17* | |
| 16. Difference valence | | 2-1 | | 2.454 (3) | | .48 | | 1.000 | | 1.271 | | .000 | | .031 | .08 | |
|  |  | 3-2 | |  |  |  |  |  |  |  |  | (.000; .202) | |  | .16* | |
| 17. Difference valence | | 3-2 | | 2.455 (3) | | .48 | | 1.000 | | 1.112 | | .000 | | .030 | .08 | |
|  |  | 3-1 | |  |  |  |  |  |  |  |  | (.000; .202) | |  | .07 | |
| * p < .05. DV’s = Dependent variables: Numbers represent task conditions with 1 = baseline, 2 = spontaneous, 3 = deliberate emotion condition. | | | | | | | | | | | | | | | | |

Supplementary Analysis: Path Model for Total Decision Outcomes Across Conditions

FIGURE S 1. Path model 9 with PCL-R facets predicting moral judgments and choice of actions across the experimental conditions

| Note*.* Significant predictors are illustrated as black arrows, grey arrows for DV’s indicate predictions at trend level. Age and IQ are included as covariates. |  |  |  |  |  |  |  |  |
| --- | --- | --- | --- | --- | --- | --- | --- | --- |

Sacrificial Moral Dilemmas

The moral dilemmas were presented in the following order and translated in German. Low-conflict dilemmas that were excluded for analyses are marked (*excluded).

Dilemmas Used for Condition 1 (Without Emotion Feedback)

***Crying Baby (Greene et al., 2001)***

Enemy soldiers have taken over your village. They have orders to kill all remaining civilians. You and some of your townspeople have sought refuge in the cellar of a large house. Outside you hear the voices of soldiers who have come to search the house for valuables. Your baby begins to cry loudly. You cover his mouth to block the sound. If you remove your hand from his mouth his crying will summon the attention of the soldiers who will kill you, your child, and the others hiding out in the cellar. To save yourself and the others you must smother your child to death.

Lawrence of Arabia (Greene et al., 2001)

You are the leader of a small army that consists of warriors from two tribes, the hill tribe and the river tribe. You belong to neither tribe. During the night a hill tribesman got into an argument with a river tribesman and murdered him. The river tribe will attack the hill tribe unless the murderer is put to death, but the hill tribe refuses to kill one of its own warriors. The only way for you to avoid a war between the two tribes that will costs hundreds of lives is to publicly execute the murderer by cutting off is head with your sword.

Vaccine Test (Greene et al., 2001)

A viral epidemic has spread across the globe killing millions of people. You have developed two substances in your home laboratory. You know that one of them is a vaccine, but you don’t know which one. You also know that the other one is deadly. Once you figure out which substance is the vaccine you can use it to save millions of lives. You have with you two people who are under your care, and the only way to identify the vaccine is to inject each of these people with one of the two substances. One person will live, the other will die, and you will be able to start saving lives with your vaccine.

*Hired Rapist (Greene et al., 2001)

You have been dissatisfied with your marriage for several years. It is your distinct impression that your wife no longer appreciates you. You remember how she appreciated you years ago when you took care of her after she was mugged. You devise the following plan to regain your wife’s affection. You will hire a man to break into your house while you are away. This man will tie up your wife and rape her. You, upon hearing the horrible news, will return swiftly to her side, to take care of her and comfort her, and she will once again appreciate you. (excluded)

*Smother for Dollars (Greene et al., 2001)

You are in hospital lounge waiting to visit a sick friend. A young man sitting next to you explains that his father is very ill. The doctors believe that he has a week to live at most. He explains further that his father has a substantial life insurance policy that expires at mid- night. If his father dies before midnight, this young man will receive a very large sum of money. He says that the money would mean a great deal to him and that no good will come from his father’s living a few more days. He offers you half a million dollars to go up to his father’s room and smother his father with a pillow. (excluded)

Footbridge (Greene et al., 2001; Thomson, 1986)

A runaway trolley is heading down the tracks toward five workmen who will be killed if the trolley proceeds on its present course. You are on a footbridge over the tracks, in between the approaching trolley and the five workmen. Next to you on this footbridge is a stranger who happens to be very large. The only way to save the lives of the five workmen is to push this stranger off the bridge and onto the tracks below where his large body will stop the trolley. The stranger will die if you do this, but the five workmen will be saved.

Dilemmas Used for Condition 2 (With Spontaneously Processed Emotion Feedback)

Submarine (Greene et al., 2001)

You are the captain of a military submarine travelling underneath a large iceberg. An onboard explosion has caused you to lose most of your oxygen supply and has injured one of your crew who is quickly losing blood. The injured crew member is going to die from his wounds no matter what happens. The remaining oxygen is not sufficient for the entire crew to make it to the surface. The only way to save the other crew members is to shoot dead the injured crew member so that there will be just enough oxygen for the rest of the crew to survive.

Ecologist (Greene et al., 2001)

You are part of a group of ecologists who live in a remote stretch of jungle. The entire group, which includes eight children, has been taken hostage by a group of paramilitary terrorists. One of the terrorists takes a liking to you. He informs you that his leader intends to kill you and the rest of the hostages the following morning. He is willing to help you and the children escape, but as an act of good faith he wants you to kill one of your fellow hostages whom he does not like. If you refuse his offer all the hostages including the children and yourself will die. If you accept his offer then the others will die in the morning but you and the eight children will escape.

*Architect (Greene et al., 2001)

You are a young architect visiting one of your construction sites with your boss. Your boss is a despicable individual who makes everyone around him miserable including you. It occurs to you that if you were to push him off of the building you are inspecting he would fall to his death and everyone would think it was an accident. (excluded)

*Countryroad (Greene et al., 2001; Unger, 1996)

You are driving along a country road when you hear a plea for help coming from some roadside bushes. You pull over and encounter a man whose legs are covered with blood. The man explains that he has had an accident while hiking and asks you to take him to a nearby hospital. Your initial inclination is to help this man, who will probably lose his leg if he does not get to the hospital soon. However, if you give this man a lift, his blood will ruin the leather upholstery of your car. (excluded)

*Transplant (Greene et al., 2001)

You are a doctor. You have five patients, each of whom is about to die due to a failing organ of some kind. You have another patient who is healthy. The only way that you can save the lives of the first five patients is to transplant five of this young man’s organs (against his will) into the bodies of the other five patients. If you do this, the young man will die, but the other five patients will live.

Burning Building (Moore et al., 2008; see also Kahane et al., 2015)

You and five other people are trapped in a burning building. There is only one emergency exit through which all of you could escape to safety, but it is blocked by burning debris. You notice another person in the hallway leading to the exit who has been injured but is about to crawl to safety through a small hole at the bottom of the exit door. You and the five people behind you do not have time to climb through the small hole. You realize that you could grab the injured man and use his body as a battering-ram to break through the burning blockage in the hallway that is preventing your escape. Doing this is certain to kill him. However, if you do not do this, you and the five people behind you will die.

Bike Week (Moore et al., 2008; see also Kahane et al., 2015)

You are an expert motorcycle rider and you have gone on vacation in order to participate in Bike Week. Thousands of other motorcycle riders from across the country have come to ride in this event. As you are riding down the road in the front of a large group of other riders you see that someone up ahead is losing control of their bike. As you speed up to pull alongside the unstable rider, you realize that this person is going to crash at any second. This would certainly result in a large pile-up and several deaths as the riders behind you run over each other trying to avoid the crashed rider. You realize that you could physically run this rider off the road and into some trees. This would cause him to crash and, at your current speed, almost certainly die, but it would prevent a crash in the middle of the street and the large pile-up of riders behind you.

Sacrifice (Greene et al., 2001)

You, your wife, and your four children are crossing a mountain range on your return journey to your homeland. You have inadvertently set up camp on a local clan’s sacred burial ground. The leader of the clan says that according to the local laws, you and your family must be put to death. However, he will let yourself, your husband, and your three other children live if you yourself will kill your oldest son.

Dilemmas Used for Condition 3 (With Deliberately Processed Emotion Feedback)

Bomb 2 (Greene et al., 2001)

You are negotiating with a powerful and determined terrorist who is about to set off a bomb in a crowded area. Your one advantage is that you have his teen-age son in your custody. There is only one thing that you can do to stop him from detonating his bomb, which will kill thousands of people if detonated. To stop him, you must contact him over the satellite hook-up that he has established and, in front of the camera, break one of his son’s arms and then threaten to break the other one if he does not give himself up.

Lifeboat 2 (Greene et al., 2001)

You are on a cruise ship when there is a fire on board, and the ship has to be abandoned. The lifeboats are carrying many more people than they were designed to carry. The lifeboat you’re in is sitting dangerously low in the water—a few inches lower and it will sink. The seas start to get rough, and the boat begins to fill with water. If nothing is done it will sink before the rescue boats arrive and everyone on board will die. However, there is an injured person who will not survive in any case. If you throw that person overboard the boat will stay afloat and the remaining passengers will be saved.

Sophie’s Choice (Greene et al., 2001)

It is wartime and you and your two children, ages eight and five, are living in a territory that has been occupied by the enemy. At the enemy’s headquarters is a doctor who performs painful experiments on humans that inevitably lead to death. He intends to perform experiments on one of your children, but he will allow you to choose which of your children will be experimented upon. You have twenty-four hours to bring one of your children to his laboratory. If you refuse to bring one of your children to his laboratory he will find them both and experiment on both of them.

*Plane Crash (Greene et al., 2001)

Your plane has crashed in the Himalayas. The only survivors are yourself, another man, and a young boy. The three of you travel for days, battling extreme cold and wind. Your only chance at survival is to find your way to small a village on the other side of the moun- tain, several days away. The boy has a broken leg and cannot move very quickly. His chances of surviving the journey are essentially zero. Without food, you and the other man will probably die as well. The other man suggests that you sacrifice the boy and eat his remains over the next few days. (excluded)

*Hard Times (Greene et al., 2001)

You are the head of a poor household in a developing country. Your crops have failed for the second year in a row, and it appears that you have no way to feed your family. Your sons, ages eight and ten, are too young to go off to the city where there jobs, but your daugh- ter could fare better. You know a man from your village who lives in the city and who makes sexually explicit films featuring small children such as your daughter. He tells you that in one year of working in his studio your daughter could earn enough money to keep your family fed for several growing seasons. (excluded)

Preventing Ebola (Moore et al., 2008; see also Kahane et al., 2015)

You are a Peace Corps health-worker who is volunteering in a rural African village. A prominent man from a nearby village has contracted an Ebola virus that is extremely contagious, incurable, and almost always fatal within a week. Miraculously, this man has survived for a month, and so he must be a rare carrier who is immune to the virus’ deadly effects. However, this man wrongly believes that your health center can cure his disease. You see him approaching and you know that if he enters the village he will spread the virus to hundreds of innocent people who, unlike him, will die. There is a loaded gun in the health center. You realize that the only way you can prevent him from entering the village and spreading the virus to you and the rest of the village is to shoot and kill him as he approaches.

Euthanasia (Greene et al., 2001)

You are the leader of a small group of soldiers. You are on your way back from a completed mission deep in enemy territory when one of your men has stepped in trap that has been set by the enemy and is badly injured. The trap is connected to a radio device that by now has alerted the enemy to your presence. They will soon be on their way. If the enemy finds your injured man they will torture him and kill him. He begs you not to leave him behind, but if you try to take him with you your entire group will be captured. The only way to prevent this injured soldier from being tortured is to shoot him yourself.

Vitamins (Greene et al., 2001; Unger, 1996)

You are the leader of a mountaineering expedition that is stranded in the wilderness. Your expedition includes a family of six that has a genetically caused vitamin deficiency. A few people’s kidneys contain large amounts of this vitamin. There is one such person in your party. The only way to save the lives of the six members of this family is to remove one of this man’s kidneys so that the necessary vitamins may be extracted from it. The man will not die if you do this, but his health will be compro- mised. The man is opposed to this plan, but you have the power to do as you see fit.

SUPPLEMENTARY REFERENCES

Greene, J. D., Sommerville, R. B., Nystrom, L. E., Darley, J. M., & Cohen, J. D. (2001). An fMRI Investigation of emotional engagement in moral judgment. *Science*, *293*(5537), 2105–2108. https://doi.org/10.1126/science.1062872

Kahane, G., Everett, J. A. C., Earp, B. D., Farias, M., & Savulescu, J. (2015). “Utilitarian” judgments in sacrificial moral dilemmas do not reflect impartial concern for the greater good. *Cognition*, *134*, 193–209. https://doi.org/10.1016/j.cognition.2014.10.005

Thomson, J. J. (1986). *Rights, restitution and risk* (W. Parent (Ed.)). Harvard University Press.

Unger, P. (1996). *Living high and letting die*. Oxford University Press.
